# Correction: Becker et al. Identifying Predictive Biomarkers for Head and Neck Squamous Cell Carcinoma Response. *Cancers* 2023, *15*, 5597

**DOI:** 10.3390/cancers16203554

**Published:** 2024-10-21

**Authors:** Anne-Sophie Becker, Cornelius Kluge, Carsten Schofeld, Annette Helene Zimpfer, Björn Schneider, Daniel Strüder, Caterina Redwanz, Julika Ribbat-Idel, Christian Idel, Claudia Maletzki

**Affiliations:** 1 Institute of Pathology, Rostock University Medical Center, 18057 Rostock, Germany; cornelius.kluge@uni-rostock.de (C.K.); cartsen.schofeld@uni-rostock.de (C.S.); annette.zimpfer@med.uni-rostock.de (A.H.Z.); bjoern.schneider@med.uni-rostock.de (B.S.); 2 Department of Otorhinolaryngology, Head and Neck Surgery “Otto Koerner”, Rostock University Medical Center, 18057 Rostock, Germany; daniel.strueder@med.uni-rostock.de; 3 Department of Internal Medicine B, Cardiology, University Medicine Greifswald, 17475 Greifswald, Germany; caterina.redwanz@med.uni-greifswald.de; 4 Institute of Pathology, University of Luebeck, University Hospital Schleswig-Holstein, Campus Luebeck, 23538 Luebeck, Germany; julika.ribbatidel@uni-luebeck.de; 5 Department of Oto-Rhino-Laryngology & Head and Neck Surgery, University of Lubeck, University Hospital Schleswig-Holstein, Campus Luebeck, 23538 Luebeck, Germany; christian.idel@uksh.de; 6 Department of Internal Medicine, Medical Clinic III—Hematology, Oncology, Palliative Medicine, Rostock University Medical Center, 18057 Rostock, Germany; claudia.maletzki@med.uni-rostock.de

## Error in Figure

In the original publication [1], there was a mistake in Figure 3A, as published. The included legend of the survival curve on the right had a wrongly named second-line caption. The correct labeling of the subgroup (dark red line) is “FOXP3 high/CMTM6 low” instead of “FOXP3 low/CMTM6 low” (which is the caption for the royal blue line). 

The corrected Figure 3A appears below. The authors apologize for any inconvenience caused and state that the scientific conclusions are unaffected. This correction was approved by the Academic Editor. The original publication has also been updated.

## Figures and Tables

**Figure 3:**
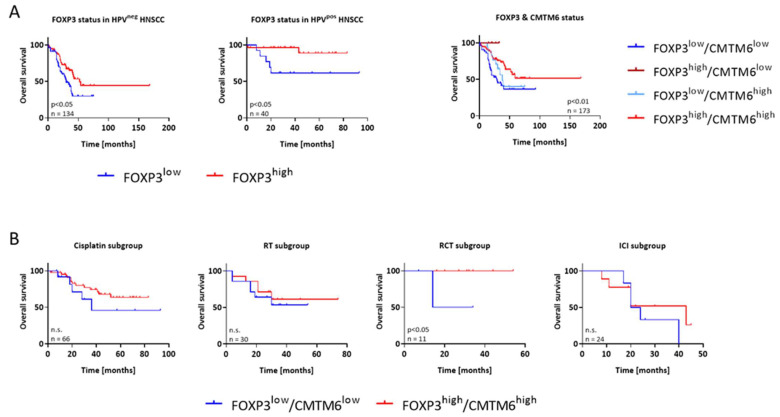
Kaplan–Meier survival curves of HNSCC patients from the discovery cohort. For biomarker-driven and treatment-related prognosis, analysis was performed on n = 177 patients receiving first- or second-line treatment. Treatment regimens included chemotherapy, radiotherapy (RT), radio-chemotherapy (RCT), or ICI (immune checkpoint inhibition). (**A**) Overall survival (OS) of patients according to FOXP3 and combined FOXP3/CMTM6 status. (**B**) Treatment-related OS stratified according to the specific regimen and FOXP3/CMTM6 status. Cisplatin-based chemotherapy, RT, RCT, or ICI. Log-rank analysis was performed to study any differences between the individual regimens. n.s.—not significant.
