# Supplementary material for: Identifying Predictive Biomarkers for Head and Neck Squamous Cell Carcinoma Response
Source: Cancers (Basel). 2023 Nov 27;15(23):5597. doi: 10.3390/cancers15235597 (PMC10705351; doi:10.3390/cancers15235597)
Supplement: Supplementary file 1 [file cancers-15-05597-s001.zip › cancers-2699178-supplementary.pdf]

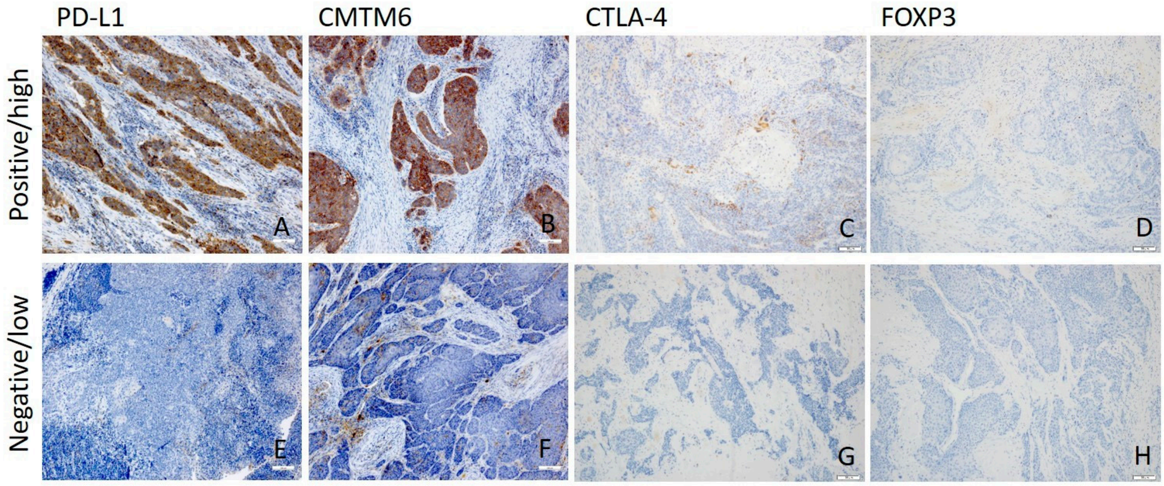

Supplementary Figure S1.: Staining for immune checkpoint molecules and immune cells in *high vs. low* expressing cases. Legend: Staining for immune checkpoint molecules and immune cells in high vs. low expressing cases: Upper row displays head and neck squamous cell carcinomas with PD-L1 positivity (A), high CMTM6 expression (B), and high status of infiltrating CTLA-4⁺ (C) and FOXP3⁺ (D) immune cells. Lower row displays head and neck squamous cell carcinomas with PD-L1 negativity (E), low CMTM6 expression (F), and low status of infiltrating CTLA-4⁺ (G) and FOXP3⁺ (H) immune cells. PD-L1 and CMTM6 assessed using the combined positive score (cut off PD-L1: ≥1; cut off CMTM6: ≥10). All 100x. Scale bar 100µm.

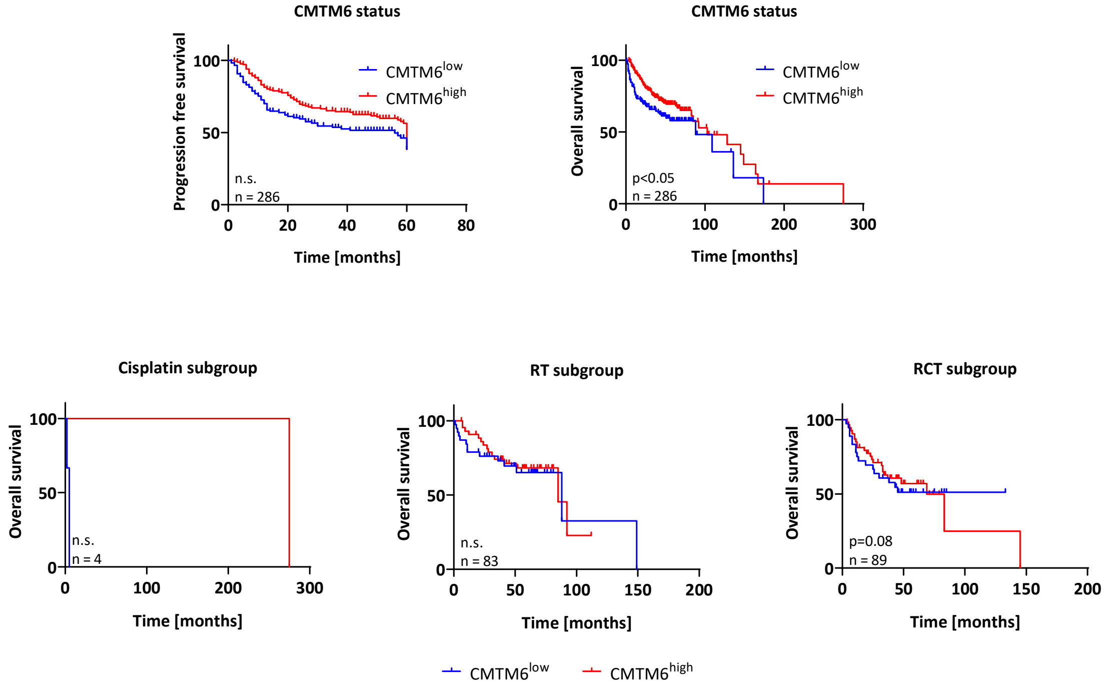

Supplementary Figure S2.: Progression-free survival and overall survival in the validation cohort stratified by CMTM6 independent of treatment and for subgroups receiving either chemotherapy, radiation or chemoradiation.
